# “Simple Rules for Editors”? Here is One Rule to Tackle Neglected Problems of Publishing

**DOI:** 10.1371/journal.pcbi.0030088

**Published:** 2007-05-25

**Authors:** Thomas C Erren, Michael Erren

The recent “Ten Simple Rules for Selecting a Postdoctoral Position” [1] complemented the Ten Rules for “Getting Published” [2], for “Writing a Grant” [3], and for “Reviewers” [4]. Together, they can do a real service for younger or less-experienced scientists to become recognized appropriately and early in their careers, be it in computational biology or in other scientific fields. We do not know which further “rules for different audiences are in the making” [2] but would like to suggest that editors or journals be a candidate audience because they are key vectors for scientific recognition. Moreover, to help editors enforce due recognition of young(er) scientists' work, we propose an Editor Rule for Appropriate Recognition: require authors to make visible who contributed substantially to the submission with ideas and work during (authorship list) or prior to (reference list) the project.

Editors, reviewers, and readers will reflexively argue that many journals, including this one, already try to document each author's contributions [1], but we feel that current practice does not suffice. Please note that a majority of young researchers in Europe and in the United States complained that they do not receive appropriate recognition of their research achievements [5,6].

Granted, journals discuss on a regular basis how important it is to give credit to authors where it's due, but we are looking at many articles concerned with who is named as author and why and where [7,8] and very few articles addressing consequences for individuals who are unduly omitted from authorship lists [9]. In addition, there is some concern that hypotheses and empirical work on which, at least part of, a publication rests are not referenced appropriately. Both these negligences can deprive mostly young(er) or less-experienced scientists of their credit, and, as a consequence, they may abandon research arenas where this happens. To avoid such dual brain drain, editors who are “strongly encouraged to develop and implement a contributorship policy” [10] should require, as a simple rule, from the first and senior (last listed) author, but preferably from all authors, the following written statements: 1) To the best of my knowledge, no one else than those individuals listed on the authorship list contributed substantially to the submitted work. And, in view of the paramount importance of ideas and hypotheses for empirical research: 2) To the best of my knowledge, author “X” on the authorship list or individual “Y” in the reference list conceived the study by having had the research idea or having put forward the study hypothesis and rationale.

Without such qualification, editors should be under no illusion: their implicit assumption that there will be no one qualifying for authorship beyond those listed and that reference lists are complete with regard to critical prior work will not be enough in numerous cases.

Importantly, a look at today's career realities evinces that entries into both the authorship and reference lists of publications are digitally monitored via MEDLINE and ISI Cited Reference Search and considered, for instance, in the process of filling open job positions and during tenure processes. Clearly, such recognition via publications is very relevant for young(er) scientists attempting to climb academic ladders. Equally clearly, editors and journals may not want to alienate contributors by our suggested statements and the resulting explicit revelations, but we need sound approaches to better rule out a largely-ignored negligence of proper recognition in published material.

In this vein, we hope that our proposal can instigate discussions as to how we can improve recognition of what young(er)—and other—scientists contribute to published research. 
